# A cross-sectional study of the relationship between daily social contact features and the prevalence of common mental disorders in Taiwan, 2000–2015

**DOI:** 10.1371/journal.pone.0312154

**Published:** 2024-10-17

**Authors:** Meng-Han Tsai, Yun-Hsuan Wu, Sih-Jie Lai, Yun-Chieh Yang

**Affiliations:** 1 Georgia Prevention Institute, Augusta University, Augusta, GA, United States of America; 2 Cancer Prevention, Control, & Population Health Program, Georgia Cancer Center, Department of Medicine, Medical College of Georgia, Augusta University, Augusta, GA, United States of America; 3 Department of Public Health, China Medical University, Taichung, Taiwan; 4 School of Chinese Medicine & Graduate Institute of Chinese Medicine, China Medical University, Taichung, Taiwan; Coventry University, UNITED KINGDOM OF GREAT BRITAIN AND NORTHERN IRELAND

## Abstract

The aim of this study was to examine the influence of daily contact features on the prevalence of common mental disorders (CMDs) in Taiwan from 2000 to 2015. Data from the Taiwan Social Change Survey for 2000, 2005, 2010, and 2015 were used to examine the relationship between the number and level of familiarity with daily social contacts with the probable CMDs (determined by score of ≥ 3 on a 12-item Chinese Health Questionnaire). Descriptive statistics and multivariable logistic regression analyses were used to assess the association. Among the 7,841 respondents, the prevalence of probable CMDs increased from 18.28% in 2000 to 21.29% in 2015. Multivariable analysis showed that respondents with more daily social contacts were less likely to have probable CMDs in the four observed years adjusting for sociodemographic characteristics and physical health limitations on daily activities in the past two weeks. A negative relationship between probable CMDs and level of familiarity with daily contacts was found in 2000 (OR = 0.67, 95% CI-0.48–0.94) and 2005 (OR = 0.70, 95% CI-0.53–0.93). Findings from our study suggest the development of culturally tailored programs/interventions through features of daily social contacts may reduce the prevalence of CMDs in Taiwan.

## Introduction

Mental health is one of rising global health concerns [1]. Particularly, mental and substance use disorders are rampant worldwide [2] and are the leading cause of years lived with disability (YLDs) [3]. Common mental disorders (CMDs) represent a broad diagnostic definition for depressive and anxiety disorders [4] and are distinct from the feelings of sadness, stress, or fear that most people occasionally experience [5]. Both depressive and anxiety disorders have a higher prevalence than other mental and substance use disorders [2], accounting for the majority of YLDs [3] as well as disability-adjusted life years (DALYs) [2]. A nationwide cross-sectional study in Taiwan showed that the prevalence of CMDs doubled between 1990 and 2010 [4]. The prevalence of treated depressive disorders increased from 1.61% in 2007 to 1.92% in 2016 according to data from Taiwan’s National Health Insurance Research Database (NHIRD) [6].

Mental health outcomes are influenced by socio-ecological factors encompassing individual, familial, and community environments, as well as broader societal structures, cultures, and beliefs across one’s lifespan [4, 7–10], which has been linked to social interaction. Social interaction (sometime used interchangeably with daily social contacts) is defined as interpersonal contact with others on the daily bases regardless of whether individuals know those people and which pathway they used [11]. Evidence has shown that daily social contact positively influences psychological well-being and mental health [12–17]. Daily social contacts may influence mental health by providing social support [12, 13, 15], improving access to material resources [18], and moderating the adverse effects of stressful life events [12, 19–22]. By interacting with other people, individuals can exchange useful information and receive material resources and emotional support, which could improve their mental health [23, 24]. In addition to general mental health, evidence has also shown the association between social interaction and depressive symptoms. A lack of social interaction has been linked to an increased risk of depression [15, 25], especially among older adults [26]. Therefore, such evidence highlights the potential influence of daily social contacts on overall mental health.

Although daily social contact is known to be related to mental health, most studies have been limited by a single point in time, small sample sizes, specific populations, a single social contact feature, and/or Western countries [27–29]. None of these particularly focus on CMDs in Taiwan. Only one study investigating the prevalence of CMDs over a 20-year period in Taiwan but without considering the influence of various daily social contacts [4]. Because daily social contacts differ from culture to culture [30, 31], the study examining how various daily social contacts (e.g., frequency or level of familiarity) impact CMDs among Taiwanese is needed. Various daily social contact features are crucial for developing an individual’s social network and represent a pivotal aspect of social action, as it contributes to the formation of network resources and social capital [32, 33]. Therefore, findings from our study may have potential for informing the development of culturally tailored interventions aimed at reducing the prevalence of CMDs through their daily social contact features. To address the gap, this study expands the research scope by examining the relationship between differential features of daily social contacts, including the number of daily contacts and levels of familiarity, and CMDs using a representative sample of Taiwan across a 15-years study period.

## Materials and methods

### Data, design, and participants

This is a secondary data analysis of the Taiwan Social Change Survey (TSCS), which was conducted by the Institute of Sociology, Academia Sinica and sponsored by the Ministry of Science and Technology, Republic of China. The TSCS is a long-term and nationally representative cross-sectional survey in Taiwan with access to social issues related to family, culture, religion, politics, social networks, and social class cyclically. The detailed methodology of the TSCS has been described elsewhere [4, 34–37]. In brief, this survey used a stratified multi-stage probability proportional to size (PPS) method to select Taiwanese citizens older than 18 years to participate in the survey. All townships and cities in Taiwan were divided into distinct clusters according to their geographic location, demographic structure, and economic situation. The sampling design involved random selection from the following three stages: 1) townships and districts, 2) lis (one of the administrative regions in Taiwan), and 3) individuals based on their respective proportional sizes. The survey is conducted by interviewers chosen by the Center for Survey Research (CSR) at Academia Sinica. These interviewers undergo training in questionnaire instructions, workflow, interviewing skills, and precautions by TSCS and CSR teams. The face-to-face data collection using a structured questionnaire was implemented by those trained interviewers at each participant’s residence address, and oral consent to participate was provided before the interviews by all participants. Ethics approval was obtained from the Institutional Review Board (IRB) of Academia Sinica.

TSCS data from the years 2000, 2005, 2010, and 2015 were used in this research and the data were accessed on October 1^st^, 2020. Data extracted for this study were publicly available and de-identified. Ethics standards for this research was approved by the Central Regional Research Ethics Committee, China Medical University, Taichung, Taiwan (CMUH REC No. CRREC-109-051). There were 1,895 respondents in 2000; 2,146 in 2005; 1,895 in 2010; and 2,034 in 2015. Out of these, 129 respondents were excluded because of missing information on age, mental health, and features of daily contacts. Ultimately, 7,841 respondents (98% of the original responses) were included in this study.

### Measures

The study outcome was common mental disorders (CMDs) measured using the 12-item Chinese Health Questionnaire (CHQ-12). The CHQ-12 is a self-reported survey included in the TSCS to evaluate respondents’ mental health every five years since 1990 and is one of the most popular and widely used screening instruments for identification and measurement of CMDs [34, 36, 38–40] (Cheng & Williams, 1986; Cheng, 1988; Cheng et al., 1990; Liu et al., 2002; Wang et al., 2004). This questionnaire not only cover direct mental health expression but also two novel aspects relevant to Chinese cultural characteristics, conveyed by somatization (the physical manifestation of psychological concerns) and family relationships. First, Cheng (1985) developed the Chinese Health Questionnaire (CHQ) by incorporating 30 items directly retrieved and translated from the General Health Questionnaire [41]. Additional 30 specifically created items aimed to be pertinent to the manifestation of CMDs in Chinese populations due to language and culture differences. Further, Cheng and Williams (1986) performed the discriminant function analysis on the CHQ to derive the CHQ-12. Scores of 0 or 1 were assigned to responses for each item in the CHQ-12, resulting in a total score ranging from 0 (better) to 12 (worse) [4, 41]. Therefore, we used the definition of total CHQ-12 scores of 3 or more as being likely to have CMDs (termed probable CMDs) and less than 3 as being unlikely to have CMDs [4, 42], which had been validated by previous research. By using this cut-off point, the sensitivity and specificity of the CHQ-12 were 87% and 77% among general medical settings in Taiwan [39] and 75% and 71% among Chinese population in Brazil [40]. In this study, the range of Cronbach’s alpha were between 0.73 and 0.79 from 2000–2015 TSCS data.

Daily contact features were our main exposures, including the number of daily contacts and level of familiarity with their daily contacts. They usually come from a direct question utilized in various large-scale social surveys that serve as a proxy measure of the size of an individual’s personal network [11]. Thus, we used a single-item survey question to measure the number of daily contacts: "How many people do you have contact with in a typical day, including all those who you say hello, chat, talk or discuss matters with, whether you do it face-to-face, by telephone, by mail or on the internet and whether you personally know the person or not?” There were six response options for the question on the number of daily contacts, including 0–4 people, 5–9 people, 10–19 people, 20–49 people, 50–99 people, and ≥ 100 people. Further, we converted each response to a single score from 1 (0–4 people) to 6 (100 or more people). Finally, the level of familiarity was evaluated by using a TSCS question, which is “How many of these people do you know?”. The responses of “almost all” and “most” were defined as a high level of familiarity and the rest responses were defined as low familiarity.

Covariates of interest were selected based on the association with probable CMDs used in previous studies [4, 43]. For sociodemographic characteristics, we included gender (male or female), age (less than 34 years, 35–49, 50–64 or 65 years or older), marital status (single, married, divorced/separated or widowed), religion (no or yes), education (elementary school or below, middle school, high school, college, university, or graduate school), employment status (employed, unemployed, retired, housewife/husband or student) and family income (score range, 1–22). Physical health limitations on daily activities were assessed using a single question, “Have you experienced any physical discomfort or injury in the past two weeks that has affected your daily activities, such as studying, working, or housekeeping?” Four response options were used, which are ranging from “no impact” to “high impact”.

### Statistical analysis

Descriptive statistics were performed to characterize probable CMDs, number of daily contacts, level of familiarity with daily contacts, sociodemographic characteristics, and physical health problems that limited daily activities during 2000–2015. Cross-tabulation of probable CMD status was also conducted to describe the differences in these characteristics by four observed data years. Chi-square tests for categorical variables and independent t-tests for continuous variables were used to examine the relationship between number of daily contacts and familiarity levels with probable CMDs. Multivariable logistic regression analyses were applied to assess the association between number of daily contacts and familiarity levels with probable CMDs, adjusted for sociodemographic characteristics and physical health limitations on daily activities in the past two weeks. The relationship between the number of daily contacts and probable CMD status modified by the level of familiarity with daily contacts was also examined in each data year. Such modification enables the explanation on whether the association between the number of daily contact and common mental disorders would vary by the level of familiarity with daily social contacts. All multivariable results were reported as odds ratios (ORs), 95% confidence intervals (CIs), and p-values. Data analyses were conducted using STATA Version 16 (Stata Corporation LLC, College Station, TX, USA). All p-values were based on two-sided probability tests. The level of statistical significance was set at 0.05.

## Results

### Participant characteristics

The distribution of features of daily contacts, sociodemographic characteristics, and physical health problems that limit daily activities are presented in **Table 1**. Of the eligible 7,841 respondents, 18.3%, 25.5%, 23.8%, and 21.3% reported having probable CMDs in 2000, 2005, 2010, and 2015, respectively. In 2010 and 2015, the average number of daily contacts was higher than that in 2000 and 2005. The results showed a significant increase from 2005 to 2010 (p-value <0.001); however, a reduction in the number of daily contacts was observed between 2010 and 2015 (p-value > 0.05). Regarding the level of familiarity with daily contacts, most respondents reported having a high level of familiarity during 2000–2015. A reduction was found from 79.8% in 2005 to 73.1% in 2010 (p-value < 0.001), and a slight increase was observed in 2015 (76.5%) (2010 vs. 2015; p-value = 0.01) among those with a high level of familiarity with daily contacts. Regarding sociodemographic characteristics, most respondents were male and aged 49 years or lower between 2000–2015. The majority of participants were married, religious, had high school education, were employed, and had no impact of physical health limitations on daily activities (**Table 1**).

**Table 1:** Participant characteristics of Taiwan Social Change Survey (TSCS) from 2000 to 2015 (n = 7,841).

		2000 (n = 1,854)		2005 (n = 2,132)		2010 (n = 1,882)		2015 (n = 1,973)	
		n	%	n	%	n	%	n	%
**Probable CMDs**									
	No	1515	81.72	1589	74.53	1434	76.20	1553	78.71
	Yes	339	18.28	543	25.47	448	23.80	420	21.29
**# of Daily Contacts**									
	1 = 0–4	155	8.36	171	8.02	121	6.43	118	5.98
	2 = 5–9	343	18.50	448	21.01	271	14.40	260	13.18
	3 = 10–19	536	28.91	629	29.50	563	29.91	587	29.75
	4 = 20–49	475	25.62	522	24.48	523	27.79	561	28.43
	5 = 50–99	198	10.68	189	8.86	221	11.74	249	12.62
	6 = ≥100	147	7.93	173	8.11	183	9.72	198	10.04
		Mean	SD	Mean	SD	Mean	SD	Mean	SD
	Score (ranged from 1–6)	3.36	1.34	3.30	1.33	3.53	1.32	3.59	1.31
**Level of familiarity**									
	Low	324	17.48	430	20.17	507	26.94	463	23.47
	High	1530	82.52	1702	79.83	1375	73.06	1510	76.53
**Gender**									
	Male	930	50.16	1065	49.95	969	51.49	1018	51.60
	Female	924	49.84	1067	50.05	913	48.51	955	48.40
**Age**									
	≤34	503	27.13	685	32.13	599	31.83	538	27.27
	35–49	743	40.08	647	30.35	532	28.27	560	28.38
	50–64	348	18.77	495	23.22	499	26.51	525	26.61
	≥65	260	14.02	305	14.31	252	13.39	350	17.74
**Marital status**									
	Single	331	17.85	561	26.33	578	30.73	586	29.75
	Married	1320	71.20	1376	64.57	1093	58.11	1127	57.21
	Divorced/separated	64	3.45	62	2.91	93	4.94	129	6.55
	Widowed	139	7.5	132	6.19	117	6.22	128	6.50
**Religion**									
	No	372	20.08	359	16.84	393	20.88	401	20.32
	Yes	1481	79.92	1773	83.16	1489	79.12	1572	79.68
**Education**									
	≤Elementary school	560	30.22	549	25.76	353	18.77	355	18.02
	Middle school	261	14.09	251	11.78	210	11.16	254	12.89
	High school	509	27.47	583	27.36	518	27.54	523	26.55
	College	282	15.22	317	14.88	278	14.78	234	11.88
	University	191	10.31	346	16.24	425	22.59	477	24.21
	Graduate school	50	2.70	85	3.99	97	5.16	127	6.45
**Employment status**									
	Employed	1244	67.13	1370	64.32	1235	65.62	1354	68.63
	Unemployed	100	5.40	122	5.73	126	6.70	172	8.72
	Retired	180	9.71	208	9.77	264	14.03	222	11.25
	Housewife/husband	282	15.22	289	13.57	170	9.03	159	8.06
	Student	47	2.54	141	6.62	87	4.62	66	3.35
**Family Income**									
		Mean	SD	Mean	SD	Mean	SD	Mean	SD
		8.00	4.69	8.53	5.09	8.66	5.20	9.51	5.52
**PH limitation on daily activities**									
	No impact	1468	79.18	1561	73.22	1509	80.18	1611	81.65
	Low impact	291	15.7	443	20.78	250	13.28	247	12.52
	Moderate impact	58	3.13	67	3.14	64	3.4	57	2.89
	High impact	37	2.00	61	2.86	59	3.13	58	2.94

Abbreviation: #, number; CMDs, Common Mental Disorders; NT, the New Taiwan; PH, physical health.

**Table 2** shows the differences between probable CMDs and features of daily contacts. Respondents with probable CMDs had significantly fewer daily contacts between 2000 and 2015 (p-value = 0.02 in 2000, p-value <0.001 in 2005, p-value = 0.002 in 2010, and p-value <0.001 in 2015). In 2000–2010, most participants with probable CMDs reported having a low level of familiarity with daily contacts; however, a reduction was observed from 25.2% in 2010 to 20.7% in 2015, although there were no significant differences. Moreover, the majority of participants were female, younger (aged ≤ 49 years), divorced or separated, unemployed, and had an impact of physical health limitations on daily activities among respondents with probable CMDs during 2000–2015.

**Table 2:** Distribution of daily contacts, sociodemographic factors, and physical health problem by probable CMDs, 2000–2015 TSCS^1^.

		2000					2005					2010					2015				
		No		Yes			No		Yes			No		Yes			No		Yes		
		n	%	n	%	p-value	n	%	n	%	p-value	n	%	n	%	p-value	n	%	n	%	p-value
**# of Daily Contacts**						0.07					0.002					0.006					<0.001
1 = 0–4		122	78.7	33	21.3		117	68.4	54	31.6		84	69.4	37	30.6		82	69.5	36	30.5	
2 = 5–9		277	80.8	66	19.2		309	69.0	139	31.0		192	70.8	79	29.2		180	69.2	80	30.8	
3 = 10–19		421	78.5	115	21.5		480	76.3	149	23.7		426	75.7	137	24.3		479	81.6	108	18.4	
4 = 20–49		401	84.4	74	15.6		391	74.9	131	25.1		417	79.7	106	20.3		444	79.1	117	20.9	
5 = 50–99		171	86.4	27	13.6		154	81.5	35	18.5		163	73.8	58	26.2		206	82.7	43	17.3	
6 = ≥100		123	83.7	24	16.3		138	79.8	35	20.2		152	83.1	31	16.9		162	81.8	36	18.2	
	Score (ranged from 1–6)	Mean	SD	Mean	SD		Mean	SD	Mean	SD		Mean	SD	Mean	SD		Mean	SD	Mean	SD	
		3.39	1.34	3.2	1.31	0.02	3.36	1.33	3.11	1.31	<0.001	3.59	1.31	3.36	1.33	0.002	3.64	1.29	3.38	1.36	<0.001
**Level of familiarity**						0.17					0.06					0.37					0.74
	Low	256	79.0	68	21.0		305	70.9	125	29.1		379	74.8	128	25.2		367	79.3	96	20.7	
	High	1259	82.3	271	17.7		1284	75.4	418	24.6		1055	76.7	320	23.3		1186	78.5	324	21.5	
**Gender**						<0.001					<0.001					<0.001					0.11
	Male	801	86.1	129	13.9		842	79.1	223	20.9		781	80.6	188	19.4		816	80.2	202	19.8	
	Female	714	77.3	210	22.7		747	70.0	320	30.0		653	71.5	260	28.5		737	77.2	218	22.8	
**Age**						0.91					0.32					0.84					0.005
	≤34	406	80.7	97	19.3		510	74.5	175	25.5		454	75.8	145	24.2		408	75.8	130	24.2	
	35–49	608	81.8	135	18.2		497	76.8	150	23.2		401	75.4	131	24.6		425	75.9	135	24.1	
	50–64	286	82.2	62	17.8		364	73.5	131	26.5		382	76.6	117	23.4		438	83.4	87	16.6	
	≥65	215	82.7	45	17.3		218	71.5	87	28.5		197	78.2	55	21.8		282	80.6	68	19.4	
**Marital status**						0.14					0.04					0.002					0.36
	Single	278	84.0	53	16.0		427	76.1	134	23.9		450	77.9	128	22.1		455	77.6	131	22.4	
	Married	1077	81.6	243	18.4		1034	75.1	342	24.9		840	77.1	250	22.9		899	79.8	228	20.2	
	Divorced/separated	55	85.9	9	14.1		40	64.5	22	35.5		56	60.2	37	39.8		95	73.6	34	26.4	
	Widowed	105	75.5	34	24.5		88	66.7	44	33.3		87	74.4	30	25.6		102	79.7	26	20.3	
**Religion**						0.45					0.06					0.04					0.85
	No	309	83.1	63	16.9		282	78.6	77	21.4		315	80.2	78	19.8		317	79.1	84	20.9	
	Yes	1205	81.4	276	18.6		1307	73.7	466	26.3		1119	75.2	370	24.8		1236	78.6	336	21.4	
**Education**						0.19					<0.001					0.67					0.71
	≤Elementary school	448	80.0	112	20.0		361	65.8	188	34.2		267	75.6	86	24.4		289	81.4	66	18.6	
	Middle school	204	78.2	57	21.8		182	72.5	69	27.5		156	74.3	54	25.7		203	79.9	51	20.1	
	High school	418	82.1	91	17.9		438	75.1	145	24.9		391	75.5	127	24.5		411	78.6	112	21.4	
	College	242	85.8	40	14.2		258	81.4	59	18.6		208	74.8	70	25.2		181	77.4	53	22.6	
	University	161	84.3	30	15.7		281	81.2	65	18.8		337	79.3	88	20.7		372	78.0	105	22.0	
	Graduate school	41	82.0	9	18.0		68	80.0	17	20.0		74	76.3	23	23.7		96	75.6	31	24.4	
**Employment status**						0.02					0.02					0.03					0.06
	Employed	1020	82.0	224	18.0		1023	74.7	347	25.3		953	77.2	282	22.8		1069	79.0	285	21.0	
	Unemployed	83	83.0	17	17.0		81	66.4	41	33.6		83	65.9	43	34.1		121	70.3	51	29.7	
	Retired	159	88.3	21	11.7		162	77.9	46	22.1		200	75.8	64	24.2		180	81.1	42	18.9	
	Housewife/husband	214	75.9	68	24.1		205	70.9	84	29.1		126	74.1	44	25.9		129	81.1	30	18.9	
	Student	38	80.9	9	19.1		116	82.3	25	17.7		72	82.8	15	17.2		54	81.8	12	18.2	
**Family Income**		Mean	SD	Mean	SD		Mean	SD	Mean	SD		Mean	SD	Mean	SD		Mean	SD	Mean	SD	
		8.13	4.77	7.42	4.27	0.01	8.87	5.11	7.55	4.88	<0.001	8.74	5.17	8.41	5.27	0.26	9.71	5.53	8.8	5.41	0.008
**PH limitation on daily activities**						<0.001					<0.001					<0.001					<0.001
	No impact	1286	87.6	182	12.4		1287	82.4	274	17.6		1244	82.4	265	17.6		1352	83.9	259	16.1	
	Low impact	182	62.5	109	37.5		261	58.9	182	41.1		150	60.0	100	40.0		139	56.3	108	43.7	
	Moderate impact	30	51.7	28	48.3		23	34.3	44	65.7		21	32.8	43	67.2		34	59.6	23	40.4	
	High impact	17	45.9	20	54.1		18	29.5	43	70.5		19	32.2	40	67.8		28	48.3	30	51.7	

Abbreviation: #, number; CMDs, Common Mental Disorders; TSCS, Taiwan Social Change Survey; NT, the New Taiwan; PH, physical health. ^1^Chi-square tests for categorical variables and t-test for continuous variables were used.

### Association between daily social contacts and prevalence of CMDs

We used multivariable logistic regression to examine the association between probable CMDs and the number of daily contacts and the level of familiarity with daily contacts, adjusted for sociodemographic characteristics and physician health problems (**Table 3**). Results showed that respondents with more daily contacts as being less likely to have probable CMDs in 2000 (OR = 0.89, p-value = 0.04), 2005 (OR = 0.88, p-value = 0.009), 2010 (OR = 0.88, p-value = 0.02), and 2015(OR = 0.85, p-value = 0.01). However, a significant relationship between probable CMDs and the level of familiarity with daily contacts was only found in 2000 (OR = 0.67, p-value = 0.02) and 2005 (OR = 0.70, p-value = 0.02). Participants with a high level of familiarity with daily contact had lower odds of probable CMDs (**Table 3**).

**Table 3:** Multivariable logistic regression to examine the association of the number of daily contacts and level of familiarity with daily contacts on probable CMDs.

		2000				2005				2010				2015			
		OR	95% CI		p-value	OR	95% CI		p-value	OR	95% CI		p-value	OR	95% CI		p-value
**# of Daily Contacts**		0.89	0.80	1.00	0.04	0.88	0.80	0.97	0.009	0.88	0.79	0.98	0.02	0.85	0.76	0.95	0.01
**Level of familiarity**																	
	Low	(reference)				(reference)				(eference)				(reference)			
	High	0.67	0.48	0.94	0.02	0.70	0.53	0.93	0.02	0.76	0.57	1.01	0.06	1.05	0.76	1.45	0.78
**Gender**																	
	Male	(reference)				(reference)				(reference)				(reference)			
	Female	1.52	1.13	2.04	0.005	1.30	1.02	1.66	0.03	1.72	1.33	2.22	<0.001	1.01	0.76	1.33	0.96
**Age**																	
	≤34	(reference)				(reference)				(reference)				(reference)			
	35–49	0.89	0.62	1.29	0.55	0.68	0.48	0.97	0.04	0.76	0.52	1.12	0.16	0.77	0.51	1.17	0.22
	50–64	0.87	0.53	1.43	0.58	0.55	0.35	0.85	0.007	0.62	0.39	0.99	0.05	0.49	0.30	0.79	0.004
	≥65	1.00	0.53	1.91	0.99	0.39	0.22	0.69	0.001	0.44	0.23	0.85	0.01	0.45	0.23	0.89	0.02
**Marital status**																	
	Single	(reference)				(reference)				(reference)				(reference)			
	Married	1.31	0.85	2.00	0.22	1.12	0.77	1.63	0.55	1.13	0.76	1.68	0.54	1.44	0.97	2.14	0.07
	Divorced/separated	0.87	0.37	2.05	0.76	1.55	0.79	3.04	0.20	1.83	1.00	3.35	0.05	1.76	0.97	3.17	0.06
	Widowed	1.41	0.71	2.79	0.32	1.28	0.70	2.33	0.42	0.94	0.47	1.87	0.85	1.49	0.71	3.13	0.30
**Religion**																	
	No	(reference)				(reference)				(reference)				(reference)			
	Yes	1.02	0.72	1.44	0.91	1.08	0.78	1.48	0.64	1.44	1.04	2.00	0.03	1.21	0.87	1.70	0.26
**Education**																	
	≤Elementary school	(reference)				(reference)				(reference)				(reference)			
	Middle school	1.63	1.04	2.56	0.03	0.67	0.44	1.02	0.06	1.17	0.71	1.91	0.54	0.93	0.53	1.64	0.81
	High school	1.16	0.76	1.78	0.50	0.53	0.36	0.78	0.001	1.05	0.67	1.63	0.84	1.21	0.71	2.06	0.49
	College	1.01	0.60	1.70	0.96	0.39	0.24	0.63	<0.001	1.14	0.69	1.90	0.60	1.41	0.77	2.57	0.27
	University	1.24	0.69	2.22	0.47	0.41	0.25	0.67	<0.001	0.82	0.49	1.39	0.47	1.15	0.62	2.11	0.66
	Graduate school	1.48	0.61	3.60	0.38	0.60	0.30	1.19	0.14	1.22	0.61	2.44	0.57	1.51	0.73	3.10	0.26
**Employment status**																	
	Employed	(reference)				(reference)				(reference)				(reference)			
	Unemployed	0.63	0.33	1.22	0.17	0.91	0.56	1.48	0.71	1.19	0.73	1.92	0.48	1.48	0.93	2.37	0.10
	Retired	0.30	0.15	0.58	<0.001	0.55	0.34	0.89	0.02	1.07	0.67	1.72	0.78	0.94	0.55	1.61	0.81
	Housewife/husband	0.81	0.54	1.22	0.31	0.77	0.54	1.11	0.17	0.87	0.54	1.40	0.57	0.93	0.54	1.60	0.78
	Student	0.98	0.39	2.49	0.97	0.62	0.35	1.09	0.10	0.67	0.33	1.35	0.27	0.63	0.28	1.44	0.27
**Family Income**		0.98	0.95	1.02	0.27	0.97	0.95	1.00	0.03	1.00	0.97	1.03	0.90	0.96	0.94	0.99	0.01
**PH limitation on daily activities**																	
	No impact	(reference)				(reference)				(reference)				(reference)			
	Low impact	4.08	3.01	5.52	<0.001	3.29	2.57	4.22	<0.001	3.24	2.37	4.42	<0.001	4.29	3.06	6.01	<0.001
	Moderate impact	7.40	4.08	13.44	<0.001	7.06	4.04	12.34	<0.001	9.18	5.04	16.71	<0.001	3.65	1.85	7.22	<0.001
	High impact	12.91	5.87	28.38	<0.001	10.25	5.42	19.38	<0.001	11.66	6.09	22.32	<0.001	5.08	2.49	10.36	<0.001

Abbreviation: #, number; OR, Odd ratio; 95% CI, 95% confidence interval; CMDs, Common Mental Disorders; NT, the New Taiwan; PH, physical health.

In addition, our findings showed that females had greater probability of developing CMDs in 2000 (OR = 1.52, p-value = 0.02), 2005 (OR = 1.3, p-value = 0.02), and 2010 (OR = 1.72, p-value = 0.02) compared with males. Age and physical health problems imposing limitations on daily activities in the past two weeks were also associated with probable CMD status. Adults aged ≥ 65 years were less likely to have probable CMDs than younger respondents(OR = 0.39, p-value = 0.001 in 2005; OR = 0.44, p-value = 0.01 in 2010; OR = 0.45, p-value = 0.02 in 2015).Those with any impact of physical health limitation on daily activities had greater odds of having probable CMDs during 2000–2015, particularly those who reported a high impact on daily activities due to physical health problems (OR = 12.91, p-value <0.001 in 2000; OR = 10.25, p-value <0.001 in 2005; OR = 11.66, p-value <0.001 in 2010; OR = 5.08, p-value <0.001 in 2015) (**Table 3**).

Finally, we examined the relationship between the number of daily contacts and probable CMDs by the level of familiarity with daily contacts (**Table 4**). Among those with high level of familiarity with daily contacts, participants with a greater number of daily contacts had lower odds of having probable CMDs in 2010 (OR = 0.88, p-value = 0.05) and 2015 (OR = 0.78, p-value <0.001). Among respondents with low levels of familiarity with daily contacts, 2000 and 2005 data indicated that those with a greater number of daily contacts were less likely to have probable CMDs, despite the lack of statistical significance in 2000 (OR = 0.08, p-value = 0.06 in 2000; OR = 0.07, p-value = 0.003 in 2005).

**Table 4:** Multivariable logistic regression to examine the association of the number of daily contacts and probable CMDs by level of familiarity with daily contacts^1^.

	High				Low			
	OR	95% CI		p-value	OR	95% CI		p-value
2000	0.92	0.81	1.04	0.20	0.80	0.63	1.01	0.06
2005	0.93	0.83	1.04	0.18	0.74	0.61	0.90	0.003
2010	0.88	0.77	1.00	0.05	0.88	0.73	1.07	0.20
2015	0.78	0.69	0.90	<0.001	1.04	0.82	1.34	0.73

Abbreviation: OR, Odd ratio; 95% CI, 95% confidence interval; CMDs, Common Mental Disorders. ^1^All models adjusted for gender, age, marital status, religion, education, employment status, family income (NT dollar), and physical health limitation on daily activities in the past two weeks.

## Discussion

Four times cross-sectional surveys demonstrated significant differences in the prevalence of probable CMDs among a nationally representative sample of Taiwanese adults. We found that an increase in the prevalence of probable CMDs was observed from 2000 to 2010, and a slight reduction was observed in 2015, which is consistent with a previous study [4]. Evidence has shown that an increase in suicide rates had been reported since 1994, but also that a reduction has been observed since 2006 [44]. This change in suicide rates in Taiwan may explain a slight reduction in prevalence rates of probable CMDs from 25.5% in 2005 to 21.3% in 2015 in our study. A possible reason may be because suicide prevention strategies advanced by establishment of efficient networks nationwide capable of delivering related care services in Taiwan [45]. Moreover, our study found that the number of daily contacts increased from 2000 to 2015; meanwhile, the high level of familiarity with daily contacts decreased from 2000 to 2010 with a slight increase observed in 2015. This shift in level of familiarity with daily contacts may be due to the high accessibility of the Internet, expansion of social networks, and replacement of the time spent in face-to-face interactions, particularly with close friends and family [29].

In our multivariable regression analysis, we found that respondents with a higher number of daily contacts or a higher level of familiarity with daily contacts were less likely to have probable CMDs. This finding is consistent with previous studies that reported fewer daily contacts associated with loneliness and depression [23, 24, 46, 47]. Individuals who have more social support and high-quality existing relationships may have better mental health [27, 48]. A greater number of daily contacts not only provide emotional support but also provide psychological comfort when needed; consequently, it enhances overall mental well-being [12, 19–22].

We also observed that the association between the number of daily contacts and probable CMDs varied across years according to various levels of familiarity with daily contacts. People with a high level of familiarity with daily contacts and a higher number of daily contacts were less likely to have probable CMDs in 2010 and 2015. In contrast, the influence of the number of daily contacts on probable CMDs among people with low levels of familiarity with daily contacts was found in 2000 and 2005. Possible explanation may be due to the rapid development of Internet technologies. People not only meet in person but also expand their social contact features by using online platforms. This is particularly true because Internet use has significantly increased since 2006 among individuals aged 12 years or older [49]. The increase in social media use, including texting, direct messaging, and video calls with close friends and family, was also observed in Taiwan [49, 50]. This is in line with our finding on having a high level of familiarity with daily social contacts was associated with lower likelihood of prevalence of CMDs in 2010 and 2015. Having a higher level of familiarity with their contacts may play a more significant role on improving mental health despite having greater number of daily social contacts [51]. Although online interpersonal interaction can overcome the limitations of time and location and make it possible to connect with a larger number of contacts, people could be more heterogeneous within those networks, resulting in low familiarity with their contacts [52, 53] which would relate to the high risk of depression [54]. However, we were unable to further evaluate the relationship of heterogeneous network and low familiarity because TSCS survey did not collect the information on heterogeneous network. Further research examining this relationship may also be helpful.

Despite not being the main study objective, our study found an increasing prevalence of probable CMDs among women. The rise in female suicide rates in Taiwan in 2000 and 2015 due to work-life balance could partially explain our finding [55]. Further, we also observed that physical limitations were associated with greater prevalence of probable CMDs. For example, chronic illnesses such as cancer, heart disease, or diabetes were associated with the risk of depression [56]; ultimately, the physical limitations of daily activities may contribute to the likelihood of probable CMDs. Finally, another important insight from our finding is that older individuals were less likely to have probable CMDs, particularly those aged 65 years or older. The dissimilar results with prior studies may be that most older individuals were retired with less stress from work or financial needs compared to younger individuals [57, 58].

Although the association between daily social interaction and mental health has been established [27–29], research particularly evaluating the influence of various daily social contact features on CMDs in Taiwan is limited. To our best knowledge, this is the first study to examine the association between the specific features of daily contacts (e.g., number of daily contacts and level of familiarity) and the prevalence of probable CMDs. Therefore, findings from our study may inform culturally tailored interventions aimed at reducing the prevalence of CMDs through individual’s daily social contact features for Taiwanese. Integrated interventions involving their social network may also be helpful. For example, use of a socio-ecological model to addresses/integrate the cumulative and intersectional experience may have potential for reducing the likelihood of probable CMDs [4, 7–10]. Finally, the association between social interaction and loneliness has been established, meanwhile during the COVID-19 pandemic, loneliness has been found as a strong predictor of mental illness [59–61]. Further research evaluating the changes of daily social contact features on mental health that are possibly linked to the pandemic is also needed.

Despite its strengths, this study has a few limitations that should be noted. First, the research design was cross-sectional, and the temporal relationship between the prevalence of probable CMDs and daily contact features cannot be established. Second, our study only considered the number of daily contacts and level of familiarity with daily contacts, without taking into account how other aspects of social interaction with daily contacts, such as frequency of contact and social settings (such as location) affects the prevalence of probable CMDs. Third, the definition of probable CMDs was only dependent on the self-reported CHQ-12 survey, which may lead to potential interpretation bias compared to clinical diagnosis. Further, as with any self-reported survey research, study participants may have given socially acceptable responses instead of answering with genuine and accurate facts. Recall bias may also have affected the accuracy of responses. Therefore, it is possible that the influence of daily contact features on probable CMDs may be overestimated or underestimated in this study. Finally, our study only included data from 2000 to 2015, which may limit ability to generalize results to the most current timeframe. Future research including more data year with a longitudinal cohort design may further elucidate the impact of various daily social contact features on the prevalence of CMDs.

## Conclusions

Overall, the prevalence of probable CMDs has increased from 2000 to 2015 in Taiwan. Individuals with more daily contacts and/or a higher level of familiarity with daily contacts were strongly associated with decreased probable CMDs. Variations in daily contact features across different periods may highlight different influences on mental health. Because of the increase in mental illnesses on psychological wellbeing, more studies are needed to further elucidate various daily contacts features in mental health. Future research with a longitudinal study design may also benefit the development of mental health promotion/intervention.
